# Disseminated Superficial Porokeratosis: A Case Report

**DOI:** 10.7759/cureus.51736

**Published:** 2024-01-06

**Authors:** Aysha Taha, Ghadah Khormi, Laila Alali, Afnan Maashi, Ahlam Alharbi

**Affiliations:** 1 Dermatology, Dallah Hospital, Riyadh, SAU; 2 Dermatology, Jazan University, Jazan, SAU; 3 Family Medicine, Primary Health Care Center, Riyadh, SAU

**Keywords:** topical corticosteroids, calcineurin inhibitors, keratotic lesions, mevalonate pathway, porokeratosis

## Abstract

Disseminated superficial porokeratosis is a rare dermatological disorder characterized by annular keratotic lesions, presenting diagnostic challenges due to its variable clinical manifestations. The pathogenesis involves genetic predisposition and environmental factors, with mutations in the mevalonate pathway implicated. Despite its benign nature, this condition significantly impacts patients' quality of life, necessitating accurate diagnosis and effective therapeutic strategies. We present the case of a 45-year-old female with a three-year history of annular plaques on sun-exposed areas progressing to involve multiple body regions. The characteristic histopathological finding of coronoid lamellae confirmed the diagnosis of disseminated superficial porokeratosis. Treatment involved a multimodal approach, including topical corticosteroids, calcineurin inhibitors, and systemic retinoids, resulting in satisfactory clinical outcomes. Long-term follow-up emphasized the need for ongoing disease monitoring and patient education regarding sun protection. The presented case underscores the importance of recognizing characteristic histopathological features for accurate diagnosis and highlights the significance of long-term follow-up, disease monitoring, and patient education to optimize outcomes and enhance overall quality of life.

## Introduction

Disseminated superficial porokeratosis is a rare and distinctive dermatological disorder characterized by the development of multiple, annular keratotic lesions with a central atrophic core [1]. First described by Mibelli in 1893, disseminated superficial porokeratosis remains a challenging condition due to its diverse clinical manifestations and the potential for misdiagnosis [1]. The pathogenesis of disseminated superficial porokeratosis is multifactorial, involving genetic predisposition and environmental factors. Alterations in the mevalonate pathway and impaired immune regulation contribute to its development. While typically benign, disseminated superficial porokeratosis can significantly impact patients' quality of life, prompting the need for accurate diagnosis and tailored management strategies [1,2].

This case report presents a comprehensive analysis of a 45-year-old female with disseminated superficial porokeratosis, emphasizing the intricate diagnostic process, therapeutic interventions, and clinical outcomes. The report underscores the importance of a meticulous clinical approach and highlights the challenges in managing this rare porokeratotic disorder.

## Case presentation

A 45-year-old female presented to our dermatology clinic with a chief complaint of persistent skin lesions. The patient reported a gradual onset of asymptomatic, small, annular plaques with a characteristic raised border and atrophic center over the past three years. She noted the lesions initially appeared on sun-exposed areas, such as the forearms and face, but later involved the trunk and lower extremities. The patient's medical history revealed no significant comorbidities or relevant family history of dermatological conditions.

Upon detailed questioning, the patient reported a lack of response to over-the-counter topical treatments, including corticosteroids and emollients. She denied any systemic symptoms, such as fever, weight loss, or joint pain. Social history was unremarkable, with no history of excessive sun exposure or relevant occupational exposures.

**Figure 1:**
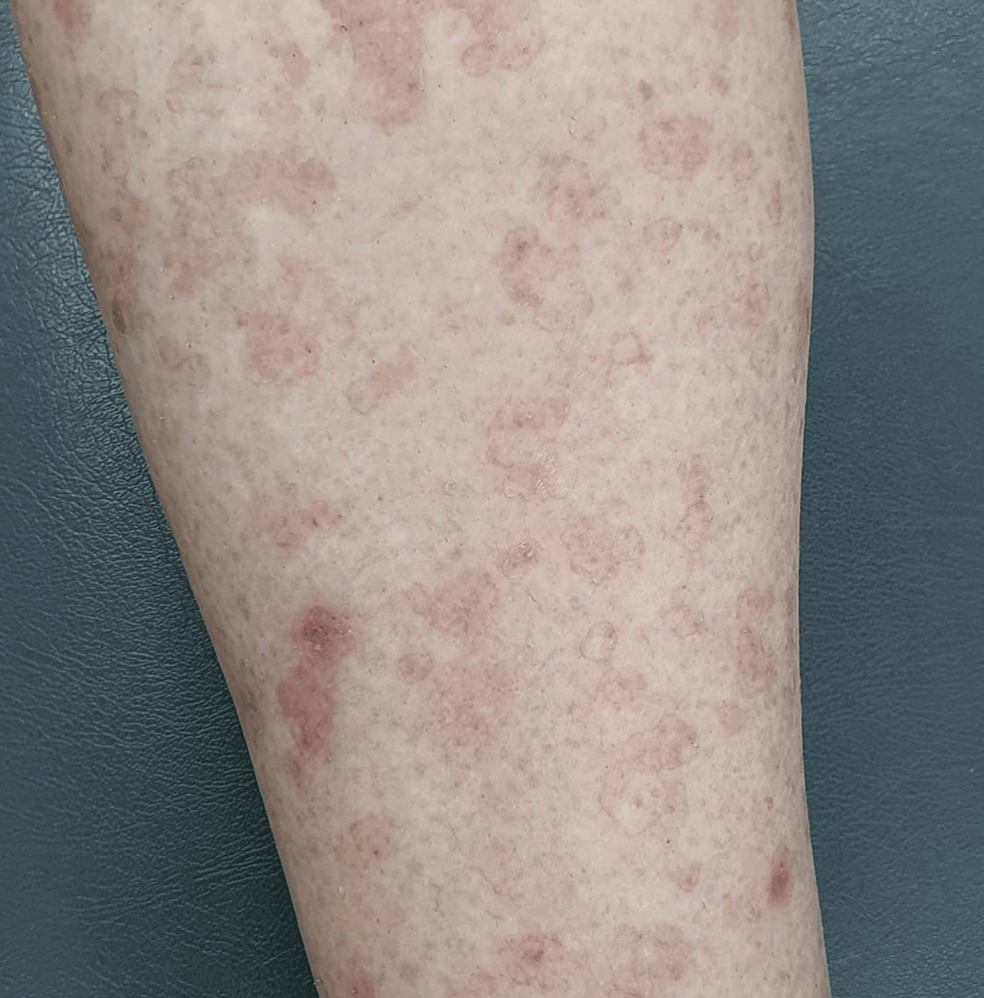
Clinical image of the lower extremity depicting characteristic annular plaques with a raised border and central atrophy, representative of porokeratosis

**Figure 2:**
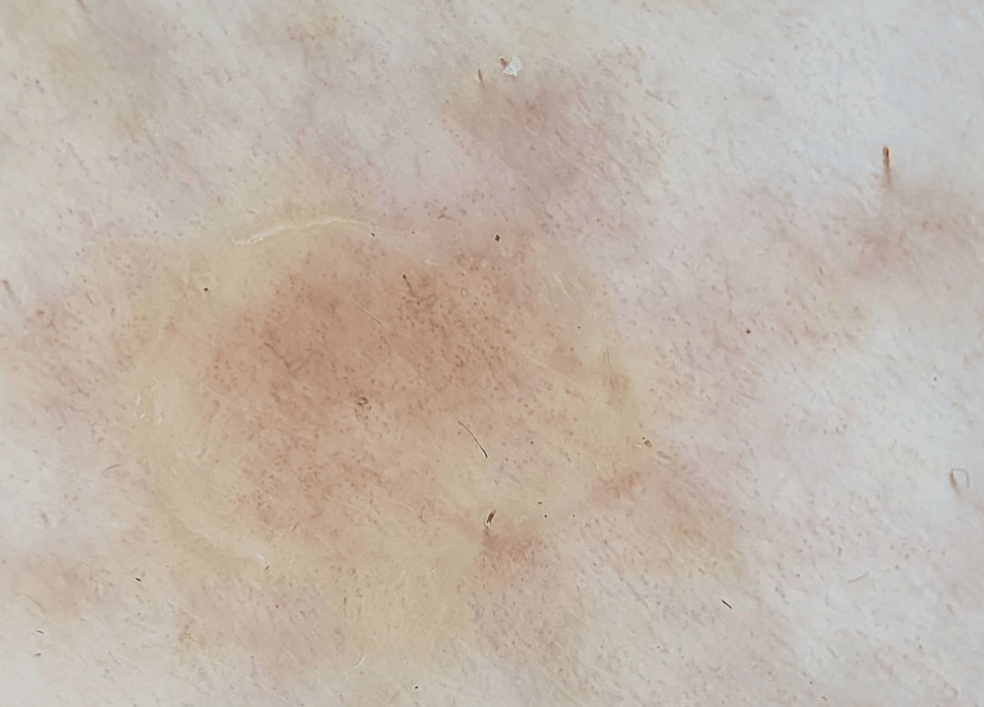
Dermatoscopic image revealing distinctive features of porokeratosis, including a coronoid lamella, supporting the clinical diagnosis of porokeratosis

Physical examination revealed multiple, well-defined, annular plaques with a raised border and central atrophy distributed symmetrically on the face, neck, upper chest, back, forearms, and lower extremities. The lesions exhibited a characteristic coronoid lamella at the periphery, suggestive of disseminated superficial porokeratosis (Figures 1-2). The differential diagnosis considered at this stage included other forms of porokeratosis, such as linear porokeratosis and punctate porokeratosis, as well as conditions presenting with annular lesions, such as tinea corporis and granuloma annulare.

**Figure 3:**
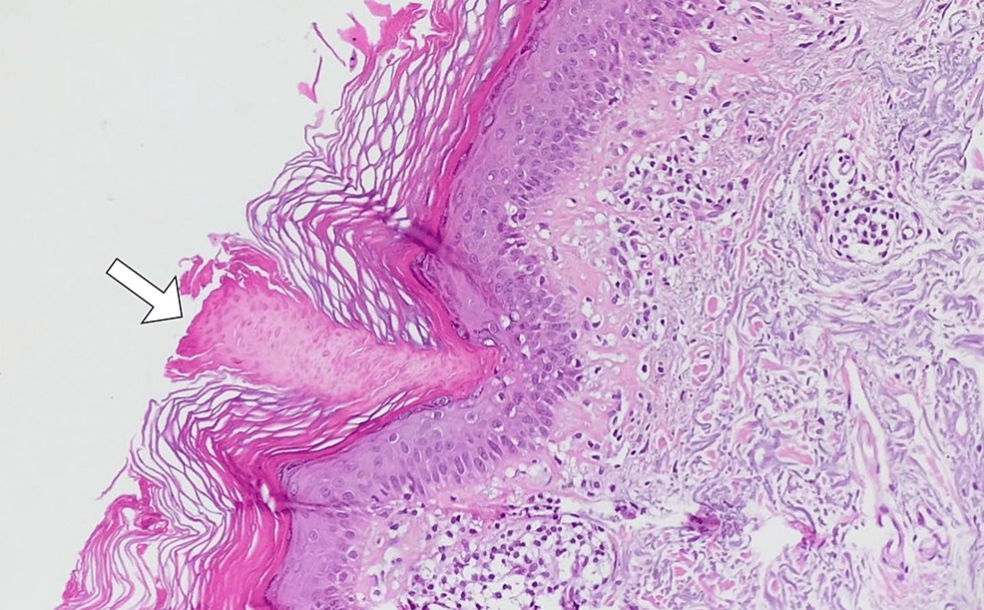
Histopathological micrograph of a skin biopsy, highlighting the presence of a coronoid lamella (arrow) confirming the diagnosis of porokeratosis

Laboratory investigations, including complete blood count, liver and renal function tests, and metabolic panel, were within normal limits (Table 1). An initial skin biopsy was performed on a representative lesion, confirming the clinical suspicion of disseminated superficial porokeratosis by revealing the characteristic coronoid lamella in the epidermis (Figure 3).

**Table 1:** Initial laboratory investigations with their reference ranges

Lab Test	Result	Reference Range
Hemoglobin	12.5 g/dL	12.0 - 15.5 g/dL
White Blood Cell Count	7,200 cells/mm³	4,000 - 11,000 cells/mm³
Platelet Count	250,000 cells/mm³	150,000 - 450,000 cells/mm³
Aspartate Aminotransferase	25 U/L	10 - 40 U/L
Alanine Aminotransferase	30 U/L	7 - 56 U/L
Alkaline Phosphatase	75 U/L	44 - 147 U/L
Total Bilirubin	0.8 mg/dL	0.2 - 1.2 mg/dL
Blood Urea Nitrogen	18 mg/dL	7 - 20 mg/dL
Creatinine	0.9 mg/dL	0.6 - 1.3 mg/dL
Glucose	90 mg/dL	70 - 100 mg/dL
Sodium	138 mEq/L	135 - 145 mEq/L
Potassium	4.2 mEq/L	3.5 - 5.1 mEq/L
Bicarbonate	26 mmol/L	23 - 29 mmol/L

The final diagnosis of disseminated superficial porokeratosis was established based on the characteristic clinical features, supported by histopathological findings. Management involved a combination of topical therapies, including corticosteroids and calcineurin inhibitors, as well as systemic retinoids to target the underlying pathogenic process.

The hospital course was uneventful, with the patient showing gradual improvement in lesion size and morphology. Regular follow-up appointments were scheduled to monitor treatment response, assess for adverse effects, and provide ongoing patient education regarding sun protection measures.

## Discussion

The presented case of disseminated superficial porokeratosis offers valuable insights into the clinical and therapeutic challenges associated with this rare dermatological disorder. Diagnostic challenges in disseminated superficial porokeratosis are well-recognized due to the condition's variable presentation and potential mimicry of other dermatoses. The characteristic histopathological finding of a coronoid lamella is crucial for definitive diagnosis [3,4]. In this case, the initial presentation on sun-exposed areas and subsequent involvement of other body regions underscore the importance of considering disseminated superficial porokeratosis in the differential diagnosis of annular lesions. The correlation between clinical features and histopathology remains pivotal for accurate identification, especially when distinguishing disseminated superficial porokeratosis from other forms of porokeratosis [1-3].

The multifactorial etiology of disseminated superficial porokeratosis involves a complex interplay of genetic predisposition and environmental triggers. Recent advancements in molecular genetics have identified mutations in the mevalonate pathway as potential contributors to the development of porokeratotic lesions [1,4]. However, further research is warranted to elucidate the precise mechanisms underlying disease pathogenesis. Investigating the genetic landscape of disseminated superficial porokeratosis may unveil potential therapeutic targets, paving the way for more targeted and effective treatment strategies [3-5].

Management of disseminated superficial porokeratosis remains challenging, with limited consensus on the optimal therapeutic approach [1]. Our case highlights the use of a combination of topical corticosteroids, calcineurin inhibitors, and systemic retinoids to achieve satisfactory clinical outcomes. This approach aligns with existing literature suggesting that a multimodal treatment strategy may be necessary for comprehensive disease control. Nonetheless, the need for long-term follow-up and the potential for disease recurrence necessitate ongoing research to refine treatment protocols and enhance patient outcomes [1,2].

## Conclusions

In conclusion, the presented case of disseminated superficial porokeratosis sheds light on the intricate clinical course and therapeutic challenges associated with this rare dermatological disorder. The multimodal treatment approach, incorporating topical corticosteroids, calcineurin inhibitors, and systemic retinoids, demonstrated satisfactory outcomes, emphasizing the importance of a comprehensive strategy in managing this condition. The diagnostic journey, marked by characteristic histopathological findings and distinctive clinical features, underscores the need for a meticulous approach to distinguishing porokeratosis from other dermatoses. The case contributes to the evolving understanding of disseminated superficial porokeratosis, emphasizing the importance of continued research to refine diagnostic criteria and optimize therapeutic interventions. As an enigmatic entity, disseminated superficial porokeratosis warrants further exploration, and this case offers valuable insights that may guide future clinical decisions and research endeavors in the field of dermatology.
